# Nucleic acid distribution pattern as a possible biomarker for metabolic activities of neoplastic cells: a digitally-aided fluorescence microscopy study on normal and neoplastic lymphocytes of acute and chronic canine lymphocytic leukemia

**DOI:** 10.1186/1475-2867-9-13

**Published:** 2009-05-11

**Authors:** Godwin N Isitor, Mervyn Campbell, Shivananda B Nayak

**Affiliations:** 1 Department of Preclinical Science, School of Veterinary Medicine, Faculty of Medical Sciences, The University of The West Indies, St Augustine Campus, Trinidad, WI, Republic of Trinidad and Tobago; 2 Department of Preclinical Science, Faculty of Medical Sciences, The University of The West Indies, St Augustine Campus, Trinidad, WI, Republic of Trinidad and Tobago

## Abstract

**Background:**

Metabolic states of neoplastic cells are increasingly being relied upon for diagnostic and prognostic assessment of neoplastic conditions. The nucleic acid distribution pattern of cells in general, in terms of degree of condensation of the nuclear chromatin and overall spread of the nucleic acid within the nuclear and cytoplasmic compartments, can reflect the metabolic state of the cell. This simple but logical concept appears not be put into consideration to date as numerous attempts are being made towards formulating reliable biomarkers for rapid diagnosis, prognosis and subsequent therapeutic interventions for neoplastic conditions. We comparatively evaluated nucleic acid distribution patterns of normal lymphocytes and neoplastic cells of lymphocytic lineage, employing light and fluorescence microscopy procedures, as well as digital imaging analytical methods.

**Results:**

The results demonstrate distinctiveness in the pattern of nucleic acid distribution for the normal lymphocytes and three lymphocytic neoplastic cell-types of canine lymphocytic leukemia that are categorized as small, intermediate and large neoplastic lymphocytes. Variably-shaped cytoplasmic processes laden with single-stranded nucleic acids (SSNA) were observed for the small and intermediate-sized neoplastic lymphocytes, compared with large neoplastic lymphocytes and the normal lymphocytes; the latter two categories of cells being virtually devoid of similar processes. Prominent cytoplasmic and nuclear clumps of SSNA, indicative of a higher rate of metabolic activity, were also observed within the neoplastic cells compared with fewer and narrower SSNA of the normal cells.

**Conclusion:**

The comparative relative increases of SSNA in cytoplasmic processes and other cellular areas of small and intermediate-sized neoplastic lymphocytes is reflective of greater metabolic activity in neoplastic cells in general compared with their normal cellular counterparts.

## Background

Nucleic acids, mostly in the form of deoxyribonucleic acid (DNA) and ribonucleic acid (RNA), constitute a major structural framework of the eukaryotic cell; the DNA being mainly distributed within the nucleus as component of the chromatin, while RNA abound within nuclear and cytoplasmic compartments. Depending on the metabolic state of the cell, the chromatin morphology varies between a more condensed form (heterochromasia) to a loosely organized or euchromatic state. In either state, the basic chromatin organization is depicted by the "beads-on-a-string" morphology, with the DNA molecules wound around the nucleosome core of 8 histone molecules [1]. The degree of tightness of the DNA molecules around the histone core determines the level of heterochromasia or euchromasia, and in turn chromatin accessibility or permissivity. A non-neoplastic cell with normal metabolic machinery can be expected to express lower DNA transcriptional activity relative to its neoplastic counterpart with a higher degree of divisional activities, which in turn translates to variability of chromatin morphology and the cellular nucleic acid architecture in general. This variability is the basis for consideration of the nucleic acid morphological state as a possible biomarker in the present investigation, utilizing canine lymphocytic leukemia (CLL) cells as a model.

CLL is of general interest, not only from the standpoint of canine health management, but also because of their striking resemblance to human lymphocytic leukemia [2,3]. CLL is relatively quite prevalent in the canine population worldwide, and the cell types appear not to have been properly elucidated to date. Existing literature commonly described two types of canine leukemia cells as small and large lymphoid types. Some authors acknowledged uncertainties with respect to terminologies and classifications of the cell types [4,5]. Given present day strides in imaging modalities for improved histological and cytological tissue processing, there is a need for a closer evaluation of the cell mix of CLL. In the past, pathologists and cytologists have relied heavily on nuclear morphologic attributes of cells in classifications of neoplasms in general. While distinctiveness of the nuclear chromatin pattern of certain cells cannot be doubted, the limitations of the use of such morphologic features under routine light microscopy with common stains, such as Wright's, Giemsa or combination of both, are enormous.

The application of digitally-aided fluorescence microscopy in diagnostic cytology appears to be gaining momentum in recent times. The use of incident light or epifluorescence microscopes, coupled with versatile fluorochromes, such as acridine orange, appears to offer great promise towards resolving issues of specific cell identity in cytological preparations; issues mostly based on both nuclear and cytoplasmic nucleic acid distribution patterns. Of particular interest is the ability to relate metabolic states of neoplastic cells to prognosis for the condition, based on the degree of associated nucleic acid fluorescence [6]. In the light of the present day paradigm shift in identification of biomarkers in cells and tissues that can aid in diagnostic, prognostic and therapeutic interventions for neoplastic conditions, there is a need to explore options other than prevailing biochemical and molecular based biomarkers. Some of the proven biomarker technologies, such as microarray profiling for DNA methylation status, involve the use of very expensive real time PCR equipment, far beyond the budget of most diagnostic setups in developing countries. Our present report represents one such investigation in which comparative digital analysis of image pixel distribution pattern of cell types of canine acute and chronic leukemia, as well as normal canine lymphocytes, can relate to their variable metabolic states and possibly serve as a reliable biomarker for diagnostic and prognostic purposes. It is also hoped that insight gleaned from such investigation would encourage greater application of digitally-aided fluorescence microscopy by pathologists in routine diagnostic and prognostic cytology.

## Results

### Morphologic Attributes based on Light and Fluorescence Microscopy

**Figure 1:**
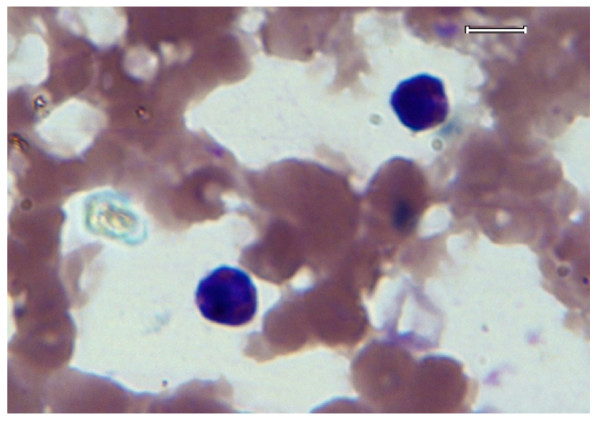
**Thin blood smear from a normal dog, stained with Wright's-Giemsa stain**. Two small lymphocytes are evident within the smear. Image was acquired at 100× objective lens magnification. Bar = 6 μm.

**Figure 2:**
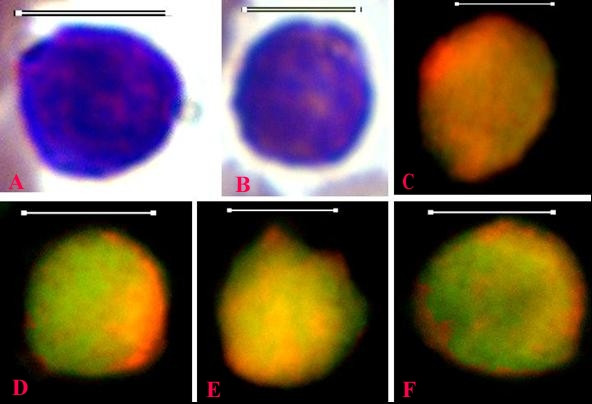
**Thin blood smear images of normal lymphocytes from healthy dogs; Wright-Giemsa stain (A, B); fluorescence microscopy images, stained with acridine orange (C, D, E, F) (Capture_6-12-05, Capture_8-12-05)**. Bar = 6 μm. The normal lymphocytes, as evidenced in panels A and B, have high nuclear to cytoplasmic ratio, and lack cytoplasmic processes as shown in both Wright-Giemsa and acridine orange-stained preparations. The fluorescing images show a distinct pattern of diffuse yellow-green DSNA fluorescence alongside punctate interconnected channel-like clumps of red SSNA fluorescence within the nuclear regions. Different stages of metabolic activity of the cells are reflected by variable degree of fluorescence by the cells. Narrow cytoplasmic rims of the cells are characterized by intense red SSNA fluorescence.

The lymphocytes were observed in the blood smears taken from the normal dog, stained with the Wright's-Giemsa stain, as sparsely distributed spherical-to-ovoid cells with a high nuclear-cytoplasmic ratio (Figure 1, Figure 2A, B). They had a fairly smooth outline with no remarkable processes; this feature is applicable to both observed large and small normal lymphocytes. The normal small lymphocytes had an average diameter of 7 μm. Under the fluorescence microscope, the normal lymphocytes in smears from normal dogs presented variable fluorescent features generally characterized by a distinct pattern of diffuse yellow-green double-stranded nucleic acid (DSNA) fluorescence, alongside punctate interconnected channel-like clumps of red single stranded nucleic acid (SSNA) fluorescence within the nuclear regions (Figure 2C, D, E, F).

**Figure 3:**
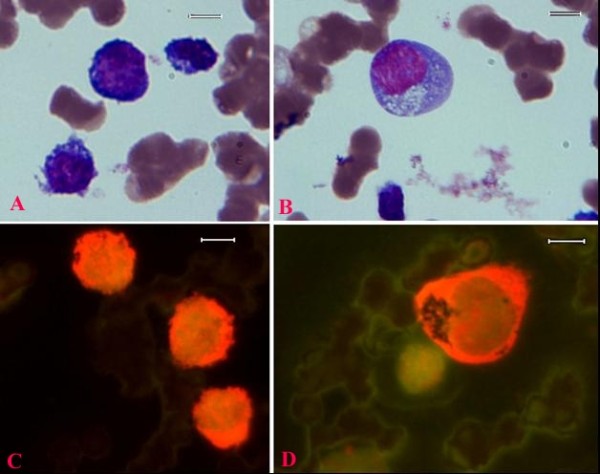
**Wright-Giemsa-stained blood smear images (A, B), and fluorescence microscopy images (C, D) of canine acute lymphocytic leukemia cells (N197605x.tif; Capture_10-11-05, Capture_14-11-05)**. The smaller neoplastic lymphocytes in panel A are equivalent to the fluorescing images in panels C, while the image of the large neoplastic lymphocyte in panel B is equivalent to the fluorescing cell in panels D. Original images were acquired at 100 objective lens magnification. Bar = 6 μm. Note the diffuse green DSNA and red SSNA fluorescence within the nuclear regions, compared to the cytoplasmic region with only red SSNA fluorescence. The smaller neoplastic cells have high nuclear to cytoplasmic ratio compared to the larger cells. Erythrocytes are devoid of any nucleic acid fluorescence.

**Figure 4:**
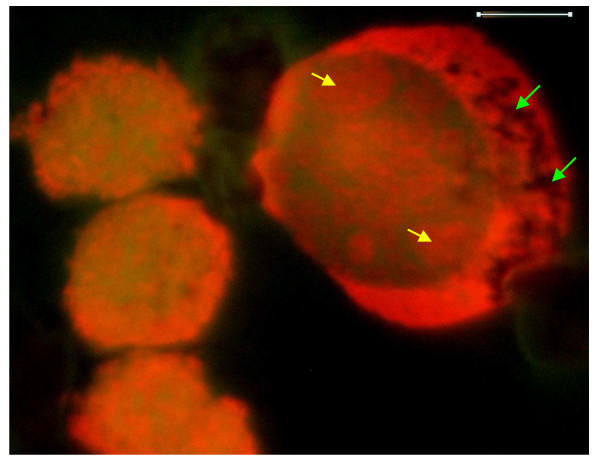
**Higher magnification of fluorescence microscopy images of the neoplastic cells, showing small and large neoplastic cells**. Original images were acquired at 100 objective lens magnification. Bar = 6 μm. Narrow cytoplasmic rim with processes laden with SSNA fluorescence are evident in the smaller neoplastic cells on the left. Cytoplasmic clumps of SSNA fluorescence are also quite evident in both cell-types. Nuclear fluorescence of the small neoplastic cells is characterized by diffuse yellow-green DSNA fluorescence with punctate, interconnected, channel-like, clumpy crimson-red SSNA fluorescence. Note the relative sizes of the small and large neoplastic cells, as well as the more oval outline of the nucleus of the large neoplastic cell which is also more eccentric, compared to the smaller cells. Nucleolar subdomains (yellow arrows) are evident in the large neoplastic cell. Non-fluorescing cisternal network (green arrows) is evident in the cytoplasm of the large cell.

**Figure 5:**
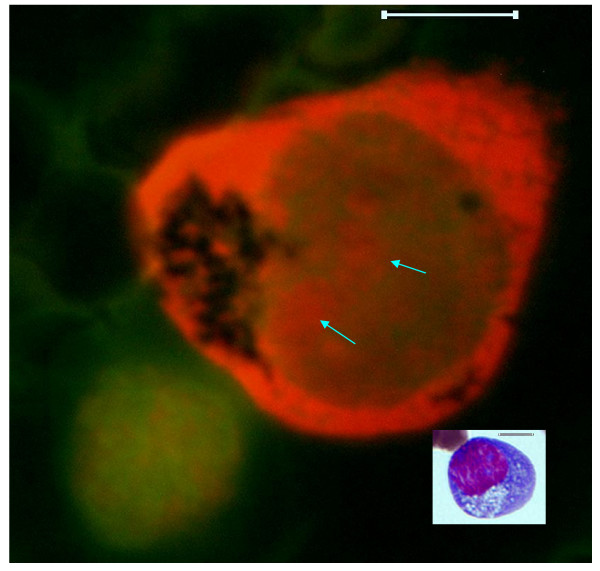
**Higher magnified fluorescence microscopy images, showing the large neoplastic cell and a normal appearing lymphocyte (Lower left)**. The insert represents equivalent large neoplastic cell prepared through routine Wright's stain, light microscopy method. Original images were acquired at 100 objective lens magnification. Bar = 6 μm. The nuclear region of the large neoplastic cell presents narrow clumps of dull green DSNA fluorescence and punctate areas of larger more diffuse clumps of red SSNA fluorescence. Nucleolar subdomain are shown as contiguous clumps of red SSNA fluorescence (Arrows). Non-fluorescing cisternal network, equivalent to the vesiculated pale structure in the Wright-stained image of the insert, is also evident in an eccentric area of the large neoplastic cell. Note the outline of the faint red SSNA fluorescence areas within the nuclear region of the lower left image of the normal-appearing lymphocyte; the distribution of which appears channel-like and interconnected. The normal-appearing cell is located close to the cytoplasmic area of the large neoplastic cell with non-fluorescing cisternal structures.

The neoplastic lymphocytes in the blood smears from the acute lymphocytic leukemia cases (Figure 3A, B), on the other hand, were more abundant within unit area of the blood smear, and were generally characterized by a more intense and diffuse nuclear and cytoplasmic crimson-red SSNA fluorescence, as well as diffuse speckled yellow-green nuclear DSNA fluorescence (Figure 3C, D, Figure 4). The smaller neoplastic lymphocytes, which measured between 7 and 8 μm in diameter, were relatively more populous compared to the large neoplastic cells which measured between 18 to 20 μm in diameter. In addition, the small cells were characterized by presence of minute irregularly-shaped cytoplasmic processes. The narrow cytoplasmic rims of the small neoplastic lymphocytes, as well as their cytoplasmic processes, were laden with clumps of diffuse crimson red SSNA fluorescence (Figure 4). A characteristic pattern of nuclear fluorescence of the small neoplastic cells was demonstrated by a diffuse yellow-green DSNA fluorescence with punctate, interconnected, channel-like, clumpy crimson-red SSNA fluorescence. The large neoplastic cells, on the other hand, typically demonstrated wider areas of clumpy diffuse red SSNA fluorescence that tended to mask fewer areas of dull-green diffuse DSNA fluorescence. Their more copious cytoplasm had diffuse clumps of the SSNA fluorescence. Non-fluorescing cisternal and vesiculated structures were also visible within the cytoplasm of the large neoplastic lymphocytes (Figure 4); the alignment of some of these structures being in unique close apposition with the eccentric nucleus (Figure 5). Diffuse red SSNA fluorescing areas attributable to nucleolar subdomain were quite prominent within nuclei of the large neoplastic lymphocytic cells (Figure 4, Figure 5).

**Figure 6:**
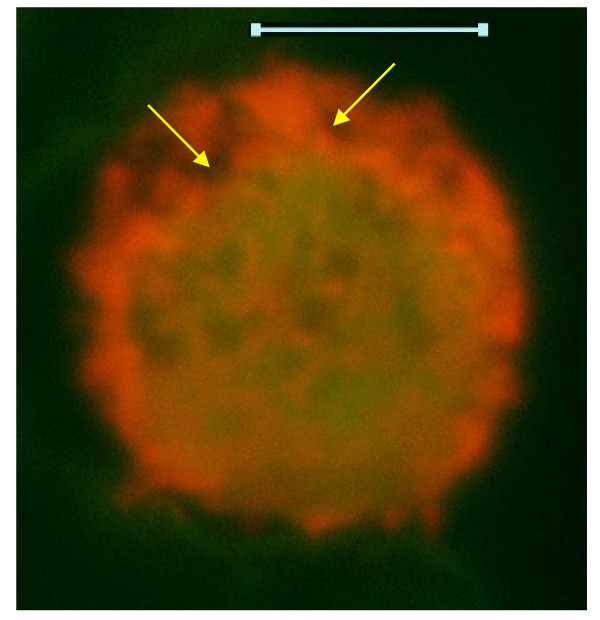
**Fluorescence microscopy image of canine acute lymphocytic leukemia blood smear (Capture_14-11-05), showing an intermediate neoplastic cell**. Bar = 6 μm. Note the presence of cytoplasmic processes, and a slightly more copious cytoplasm compared to those of the small neoplastic lymphocytes shown in Figure 4. The non-fluorescent cisternal structures are present (arrows) within the cytoplasm, but fewer than those of the large neoplastic cells. Nuclear fluorescent pattern is also similar to those of the small neoplastic cells.

**Figure 7:**
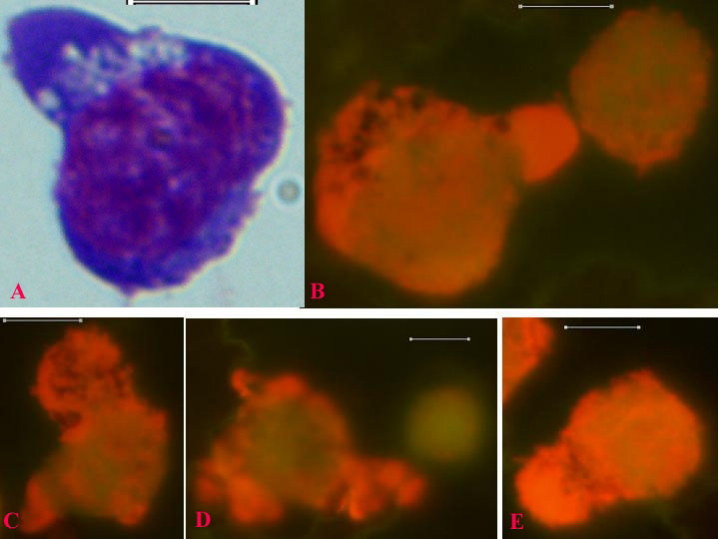
**Wright-Giemsa-stained blood smear image (A) and fluorescence microscopy images (B, C, D, E) of canine acute lymphocytic leukemia cells (Capture_10-11-05; Capture_14-11-05), showing intermediate neoplastic cells with variable cytoplasmic processes**. Original images were acquired at 100 objective lens magnification. Bar = 6 μm. A process of induction of normal appearing smaller lymphocytes by the neoplastic cells appears to be taking place (panels B and D). Chunks of cytoplasmic processes laden with SSNA materials are being extruded from some of the neoplastic cells.

Intermediate-sized neoplastic lymphocytes, ranging between 11 and 12 μm in diameter, were also observed (Figure 6, Figure 7). These cells had fluorescing features similar to those of the small neoplastic cells, and in addition had reasonably copious cytoplasm with a fewer non-fluorescing cisternal structures in comparison with those of the large neoplastic cells. The cytoplasmic processes of the intermediate neoplastic cells were variable in terms of shape and size; some of the processes tending to be chunky, and suggestive of being extruded from the cytoplasm (Figure 7). This observation was also made in the Wright-Giemsa stained smears (Figure 7A).

Normal-appearing lymphocytes were observed in close association with some of the intermediate and large neoplastic cells (Figure 5, Figure 7A, B, D). These normal appearing cells were significantly smaller than the neoplastic cells, and were generally characterized by nuclear diffuse yellow-green DSNA fluorescence and punctate red interconnected clumps of SSNA fluorescence. Some of the normal-appearing cells seem to be in direct contact with the neoplastic cells through large processes of the latter, in a manner suggestive of induction of the normal-appearing cells by the neoplastic ones (Figure 7B). These large processes were of variable sizes, and exhibited intense red SSNA fluorescence.

**Figure 8:**
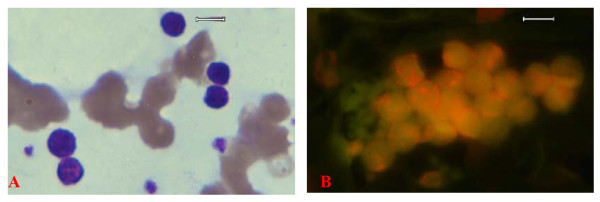
**Wright-Giemsa stained blood smear image (A), and fluorescence microscopy image (B) of canine chronic lymphocytic leukemia cells (Capture_5-6-06)**. Original images were acquired at 100 objective lens magnification. Bar = 6 μm. Note the characteristic clumping of the lymphocytes in both panels; the clumping being more pronounced in the fluorescence microscopy equivalent image (panel B).

**Figure 9:**
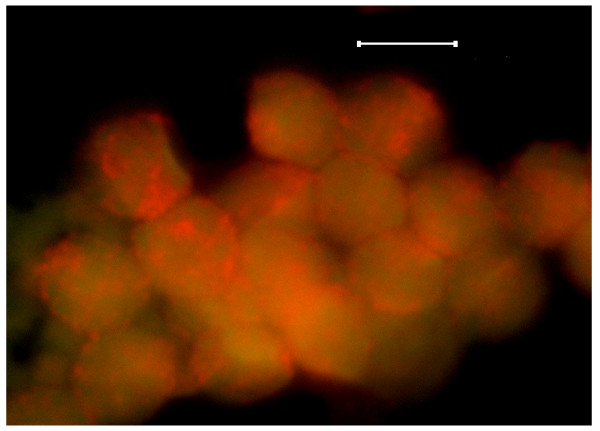
**Higher magnification of the cells shown in Figure 5B**. Original image was acquired at 100 objective lens magnification. Bar = 6 μm. Punctate areas of red SSNA fluorescence are distinct as interconnected clumps amongst the diffuse light green DSNA fluorescence in their nuclear regions.

The neoplastic lymphocytes within blood smears of the chronic lymphocytic leukemia case were observed as typically clumped cells (Figure 8, Figure 9). Each cell within the clump had features of the typical small neoplastic cell of the acute lymphocytic leukemia, in addition to the nuclear red SSNA fluorescing clumps being quite vivid amidst a more diffuse faint yellow-green DNA fluorescence.

There were, generally, no observed fluorescence attributable to the presence of mitotic figures, such as chromosomal clumping at the poles or equatorial regions of the cells in the blood smears of any of the leukemia cases.

### Digital Analytical Attributes and Statistical Features

Results of the digitally analyzed images of the different cell-types are presented in figures 10 to 22. Greater concentration of pixel intensity values, as denoted by the amplitude of the different curves of frequency distribution of pixel intensity values, were generally observed as being associated with SSNA fluorescence.

**Figure 10:**
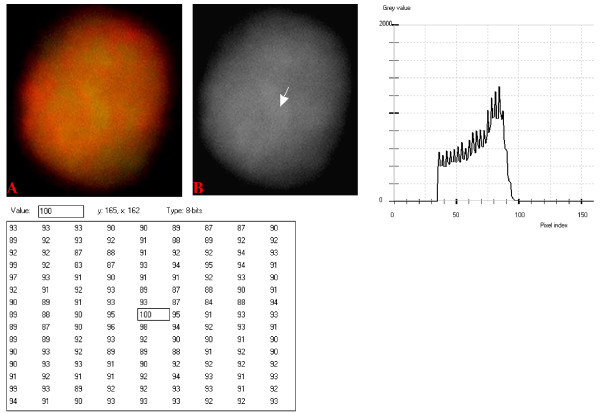
**24-bit fluorescence (A) and 8-bit gray-tone (B) images of normal dog small lymphocyte**. Subtracted 30 to remove background pixels; added 33 to set highest reference pixel point to 100 as shown by the point of the arrow in panel B corresponding to the value 100 in the adjoining Table. Note the positively sloping mountain-like curve, skewed to the right and with 17 spikes which are mostly located at the top of the curve. The sides of the curve are extremely steep.

**Figure 11:**
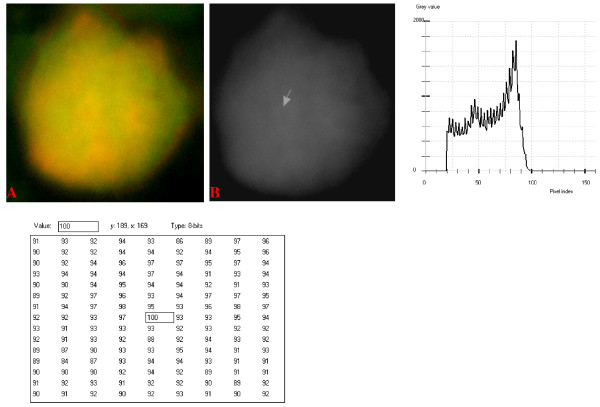
**24-bit fluorescence (A) and 8-bit gray-tone (B) images of normal dog small lymphocyte**. Subtracted 63 to remove background pixels; added 18 to set highest reference pixel point to 100 as shown by the point of the arrow in panel B corresponding to the value 100 in the adjoining Table. Note the positively sloping mountain-like curve with 23 spikes which are mostly located at the top of the curve. The sides of the curve are steep, while a set of spikes are more extruded at the middle of the curve.

**Figure 12:**
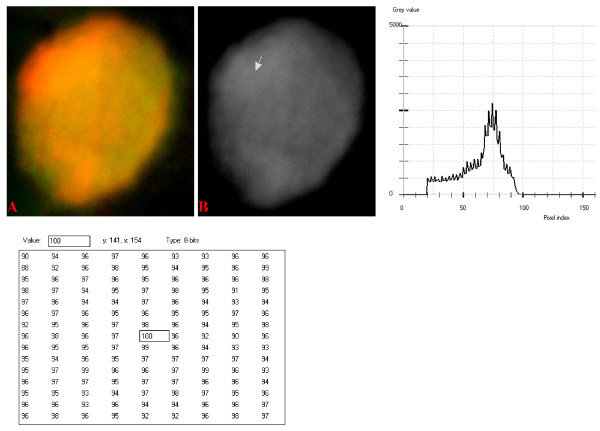
**24-bit fluorescence (A) and 8-bit gray-tone (B) images of normal dog small lymphocyte**. Subtracted 55 to remove background pixels; added 18 to set highest reference pixel point to 100 as shown by the point of the arrow in panel B corresponding to the value 100 in the adjoining Table. The curve has a fairly extended shoulder at its left side. Note 5 spikes which are mostly located at the peak of the curve, while minute spikes project from the shoulder.

**Figure 13:**
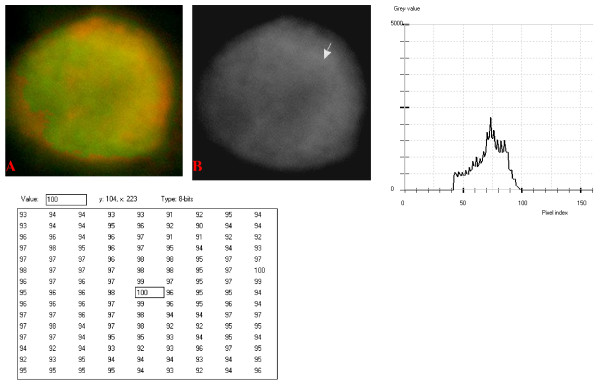
**24-bit fluorescence (A) and 8-bit gray-tone (B) images of normal dog small lymphocyte**. Subtracted 60 to remove background pixels; added 40 to set highest reference pixel point to 100 as shown by the point of the arrow in panel B corresponding to the value 100 in the adjoining Table. The curve is more mountain-like than that of Fig. 12. Note 6 spikes which are mostly located towards the peak of the curve, while minute spikes project from the shoulder.

**Figure 14:**
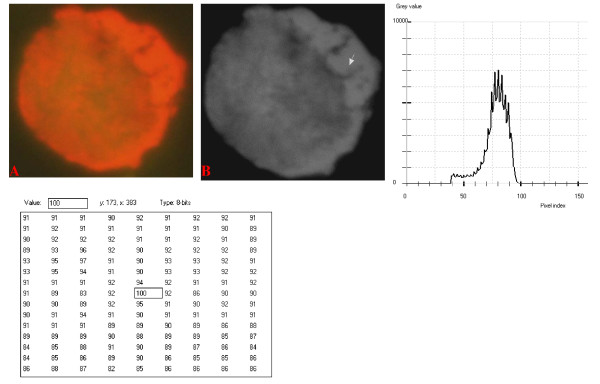
**24-bit fluorescence (A) and 8-bit gray-tone (B) images of small neoplastic lymphocyte**. Subtracted 65 to remove background pixels; added 37 to set highest reference pixel point to 100 as shown by the point of arrow in panel B corresponding to the value 100 in the adjoining Table. The mountain-like curve has an extended shoulder at its left side. Note 9 spikes are mostly located at the broad-based peak of the curve, while minute spikes project from its shoulder.

**Figure 15:**
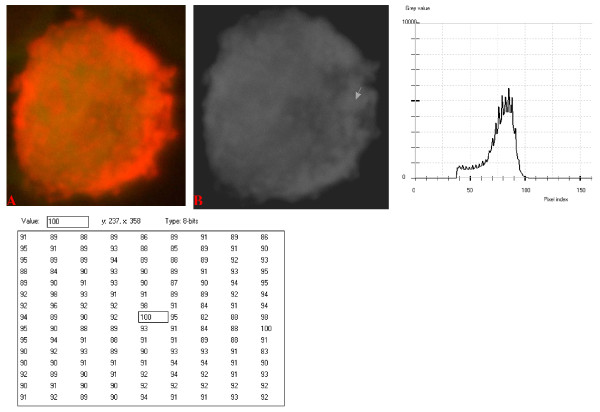
**24-bit fluorescence (A) and 8-bit gray-tone (B) images of small neoplastic lymphocyte**. Subtracted 50 to remove background pixels; added 36 to set highest reference pixel point to 100 as shown by the point of arrow in panel B corresponding to the value 100 in the adjoining Table. The curve is fairly similar to that of Fig. 14.

**Figure 16:**
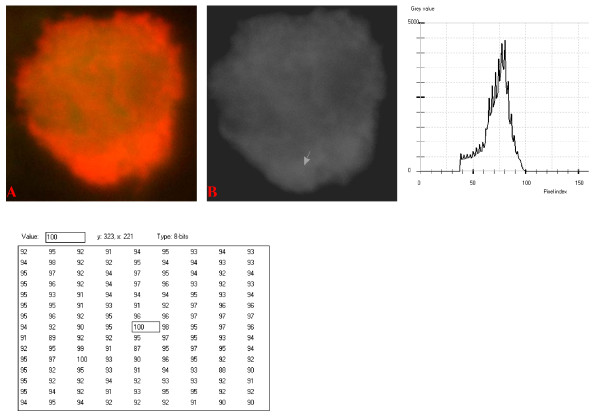
**24-bit fluorescence (A) and 8-bit gray-tone (B) images of small neoplastic lymphocyte**. Subtracted 55 to remove background pixels; added 36 to set highest reference pixel point to 100 as shown by the point of arrow in panel B corresponding to the value 100 in the adjoining Table. The curve has a narrower peak, and 9 spikes mostly at the sides of the peak. It also has a left shoulder with minute spikes.

**Figure 17:**
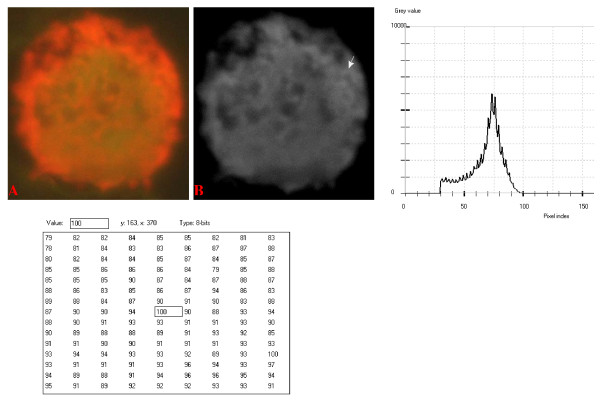
**24-bit fluorescence (A) and 8-bit gray-tone (B) images of intermediate neoplastic lymphocyte**. Subtracted 51 to remove background pixels; added 28 to set highest reference pixel point to 100 as shown by the point of arrow in panel B corresponding to the value 100 in the adjoining Table. The curve is similar to that of Fig. 16; its left shoulder with minute spikes is more extended.

**Figure 18:**
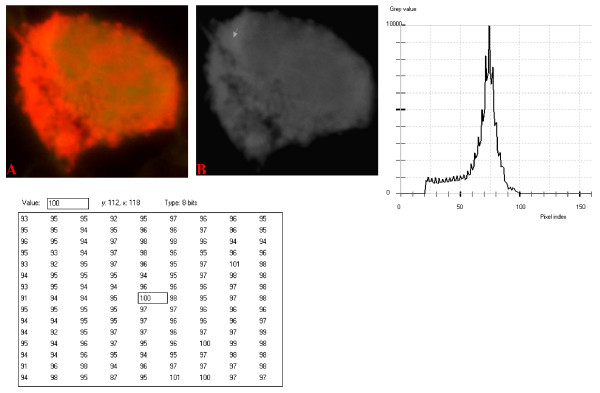
**24-bit fluorescence (A) and 8-bit gray-tone (B) images of intermediate neoplastic lymphocyte**. Subtracted 50 to remove background pixels; added 18 to set highest reference pixel point to 100 as shown by the point of arrow in panel B corresponding to the value 100 in the adjoining Table. The curve is fairly similar to that in Fig. 17.

**Figure 19:**
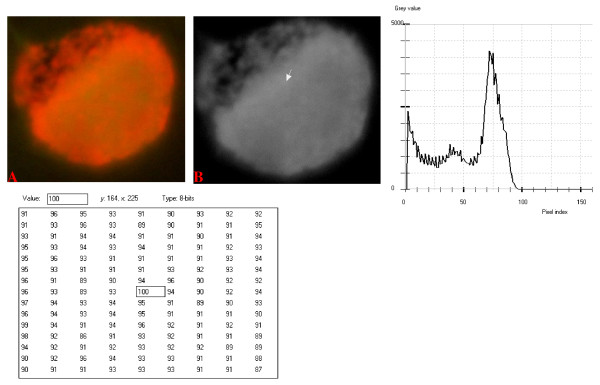
**24-bit fluorescence (A) and 8-bit gray-tone (B) images of intermediate neoplastic lymphocyte**. Subtracted 40 to remove background pixels; no addition to set highest reference pixel point to 100 as shown by the point of arrow in panel B corresponding to the value 100 in the adjoining Table. The curve has 2 main peaks flanking a trough-like region with numerous spikes. The left side of the curve is steep.

**Figure 20:**
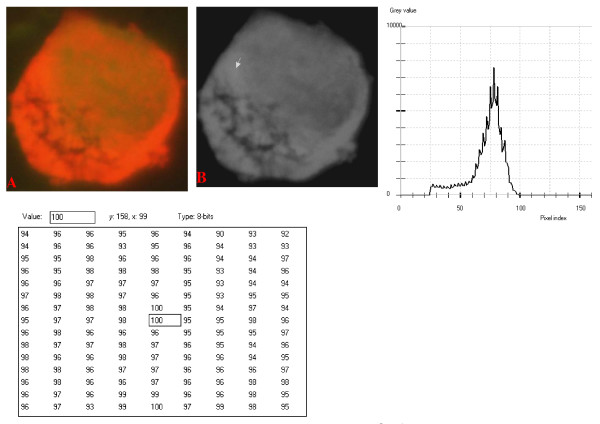
**24-bit fluorescence (A) and 8-bit gray-tone (B) images of large neoplastic lymphocyte**. Subtracted 50 to remove background pixels; added 23 to set highest reference pixel point to 100 as shown by the point of arrow in panel B corresponding to the value 100 in the adjoining Table. The curve is similar to that of intermediate neoplastic lymphocyte shown in Fig. 17 and 18. It is sloped at both sides, and has a main peak, narrowed at the top, and with 10 spikes at the sides. A left shoulder is also present.

**Figure 21:**
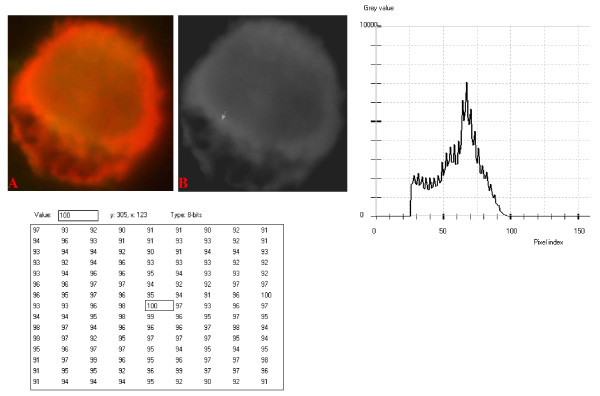
**24-bit fluorescence (A) and 8-bit gray-tone (B) images of large neoplastic lymphocyte**. Subtracted 50 to remove background pixels; added 24 to set highest reference pixel point to 100 as shown by the point of arrow in panel B corresponding to the value 100 in the adjoining Table. The curve is fairly similar to that in Fig. 20, except for more pronounced spikes at the left shoulder of the latter; the outline of the shoulder is sigmoid-like.

**Figure 22:**
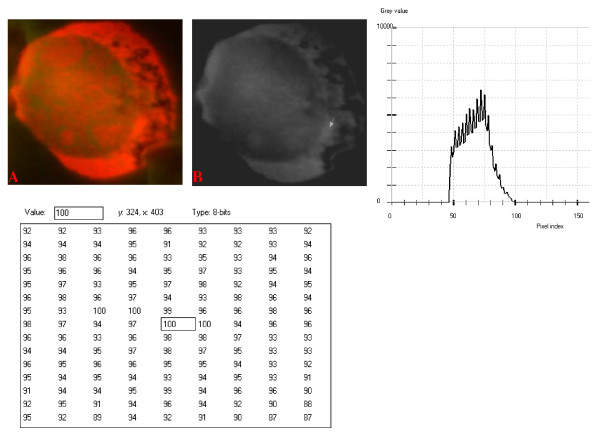
**24-bit fluorescence (A) and 8-bit gray-tone (B) images of large neoplastic lymphocyte**. Subtracted 60 to remove background pixels; added 45 to set highest reference pixel point to 100 as shown by the point of arrow in panel B corresponding to the value 100 in the adjoining Table. The curve is quite different from those of same categories (Figs. 20 and 21). It has a mountain-like appearance with 11 spikes located at the peak.

The curves for the normal lymphocytes were of variable outline, but generally tended to be either mountain-like with characteristic 17 to 23 spikes at their mountain-tops (Figure 10 and Figure 11), or with 5 to 6 spikes mostly located at the wide peak regions of the curves, while numerous minute spikes occurred at the left shoulders of the curves (Figure 12 and Figure 13). The positions of the highest pixel intensity reference point of 100 tended to correspond for the two images with mountain-like curves within their nuclei, and also for the other two images with extended curve left shoulders within their cytoplasmic areas. There was variability of the mean pixel intensity values (P < 0.05) among the images of the normal cell-type.

The curves for the small neoplastic cells (Figure 14, Figure 15 and Figure 16) were also mountain-like, but generally had narrower peak region and an extended left shoulder. Tall spikes, approximately 9 in number, projected from the tops and sides of the peak region, while numerous minute spikes radiated from the shoulder of the curves. There was a high degree of resemblance between the cytoplasmic positions of the highest pixel intensity reference points for the cells of this category. The mean pixel intensity values for the images were also variable (P < 0.05).

Curves of the intermediate neoplastic cells were of two broad types. The first type (Figure 17 and Figure 18) had close resemblance with the curves of the small neoplastic cells, except for the longer left shoulder of the former. The positions of the highest pixel intensity points for this curve-type cells were similar at the cytoplasmic regions. The second curve-type (Figure 19) was characterized by two peak regions which flanked a trough-like region with numerous minute spikes. The mean pixel intensity values for the images of this category of cells were also variable (P < 0.05).

Curves of the large neoplastic cells varied between mountain-like type with narrow peak region and extended left shoulder (Figure 20 and Figure 21), to the type that was simply mountain-like with broader peak region (Figure 22). The curve in Figure 21 appears to be a slight variant of that in Figure 20, in that its left shoulder is sigmoid-like. The minute spikes present at the left shoulder of the curve in Figure 20 are replaced by taller spikes in the curve of Figure 21. The broader-peak curve-type (Figure 22) contains approximately 11 tall spikes at the top of the peak region, while smaller spikes radiate from the steep right shoulder. The mean pixel intensity values for the images of this category of cells were also variable (P < 0.05). The positions of the highest pixel intensities also corresponded quite well at cytoplasmic areas of the cells of the category.

## Discussion

The nucleic acid distribution patterns observed for the cells in the present investigation clearly supports the existence of three lymphocytic neoplastic cell types – small, intermediate and large cells, for the canine acute lymphocytic leukemia, contrary to previously held view on the existence of only small and the large types. Note that cells of the chronic lymphocytic leukemia closely resembled the small neoplastic cells of the acute lymphocytic leukemia, except for the clustered arrangement of the cells of the former. The slightly modified Von Bertalanffy's methodology [7] for the acridine orange-stained fluorescence microscopy procedure is a well accepted and reliable method for differentiation of nucleic acids within cells. The stain has been shown to have high affinity for nucleic acids by intercalating with DSNA molecules and by binding electrostatically with SSNA molecules, respectively emitting yellow-green and red color fluorescence [8-10]. The choice of the Von Bertalanffy's method over other methods, such as Jahanmehrs's [11], stems from the issue of specificity. The alcohol fixation process with Von Bertalanffy's method leaches-out cellular inclusions such as lysosomal and secretory contents that can stain positively with acridine orange, while Jahanmehrs's method which does not involve fixation step enables cellular inclusions to also fluoresce.

The three cell-type paradigm for the canine acute lymphocytic leukemia is supported by both morphologic staining attributes as well as digital analytical features of the cells. Small, intermediate and large lymphocytes have also been demonstrated in cytological preparations of normal tissue [12]. With respect to morphologic attributes, the sizes of the respective small, intermediate and large neoplastic cells were 7 to 8 μm, 11 to 12 μm, and 18 to 20 μm. The small neoplastic cells, in addition to having high nuclear-cytoplasmic ratio, were characterized by presence of minute irregularly-shaped cytoplasmic processes, while the larger intermediate cells with more cytoplasm had more chunky, variably-sized, cytoplasmic processes (Figure 4 and Figure 6). Clumps of cytoplasmic and nuclear SSNA fluorescence were quite pronounced in both small and intermediate neoplastic cells; however, the presence of few non-fluorescing cisternal structures within the cytoplasm of the latter cells is a distinguishing feature. Nuclear DSNA fluorescence in the small and intermediate cells was similar, and characterized by diffuse areas of yellow-green fluorescence. The distinguishing morphologic features of the large neoplastic cells, apart from the larger size, bordered mostly on the more diffuse nature of the abundant cytoplasmic SSNA fluorescence, as well as presence of prominent cytoplasmic non-fluorescing cisternal structures (Figure 4 and Figure 5). The large neoplastic cells also had a smoother cytoplasmic outline virtually devoid of processes, while its nuclear fluorescence pattern was more of diffuse dull-green DSNA type with broad areas of clumpy interconnected SSNA red fluorescence. The observed non-fluorescence cisternal structures in the intermediate and large neoplastic cells appear similar to fibrillar inclusions reported for lymphoma and leukemia cells [13,4], and also similar to filaments of plasma cells of human solitary myeloma [14]. These earlier reports suggested possible secretory functions for the structures.

In comparison with the normal lymphocytes, which are mostly characterized by a distinct pattern of nuclear diffuse yellow-green DSNA fluorescence, alongside punctate interconnected narrower clumps of red SSNA fluorescence, the overall nucleic acid distribution pattern of the neoplastic cells is highly indicative of aggressive metabolic activity. The existence of prominent cytoplasmic and nuclear clumps of SSNA fluorescence within the neoplastic cells compared with the fewer and narrower SSNA fluorescence of the normal cells suggest a greater degree of metabolic activity in the neoplastic cells. Since the SSNA fluorescence is most likely due to increased RNA content, which in turn reflects higher level of nuclear DNA transcriptional activity, it can be argued that the neoplastic cells are generally more transcriptionally active compared to the normal cells. Also of immense interest is the observation of SSNA-fluorescent cytoplasmic processes and chunks in the small and intermediate cells. These processes, which are more chunky in the intemerdiate cells, appear to be extruding cytoplasmic fragments laden with nucleic acid capable of bringing about induction of normal lymphocytes into neoplastic cells. This notion is supported by the fact that some earlier reports indicate increases of the neoplastic cells in chronic lymphocytic leukemia through accumulation rather than proliferation of the cells [3,15-17]. These earlier reports, however, suggest increase life span and decreased apoptosis as probable reasons for the noticeable build-up of cells in terms of number rather than our observation which suggests an inductive process whereby normal appearing lymphocytes are converted to neoplastic cells through nucleic acid materials extruded from the neoplastic cells and possibly internalized through endocytosis by the normal cells. The lack of observation of fluorescing patterns attributable to mitotic divisional process also buttresses this view point. Although canine lymphocytic leukemia has been successfully transplanted into fetuses by means of intact cells in the past [2,18], it may be possible to carryout such transplants through the use of cell-free extracts containing cytoplasmic chunks of nucleic acids. The non-fluorescing cisternal structures, observed in the cytoplasm of the intermediate and large neoplastic cells, appear to be secretory structures of non-nucleic acid substances. Such substances may also be of inductive relevance, since normal appearing lymphocytes tended to be in close contact with the large neoplastic cells, especially at the cytoplasmic regions containing the cisternal structures (Figure 5).

Digital analytical attributes for both normal and the neoplastic cells, in terms of the features of the generated curves of frequency distribution of pixel intensity values, further explain the uniqueness of the cell-types as well as their metabolic states. Within each cell-type, the curve outlines tended to have a fair degree of resemblance, even though their mean pixel intensity values obtained from 135 clusters of pixel population at the highest pixel intensity points manifested variability through the ANOVA test, α = 0.05. The variability of the mean pixel intensity values within cell-types simply reflects variable concentration of nucleic acids which in turn denotes variable states of metabolic activity of the cells, while semblance of their curves of frequency distribution of pixel intensity values reflect similarity in nucleic acid distribution pattern in each cell-type. Of particular interest is the fact that there was a high degree of correspondence of the positions of highest pixel intensity points among cells of the same class. The cells that manifested greater similarity with respect to the positioning of the highest pixel intensity points apparently showed greater curves similarities (Figure 10 and Figure 11, Figure 14, Figure 15, Figure 16, Figure 17 and Figure 18). Subsequently, curve-shape variability as well as variability in the positions of highest pixel intensity points were mostly recorded for the large neoplastic cells (Figure 20, Figure 21 and Figure 22).

Among the normal cell-type, the corresponding positions of highest pixel intensity values were at nuclear SSNA-fluorescent points (Figure 10, Figure 11, Figure 12 and Figure 13). Those of the small neoplastic cells corresponded at cytoplasmic SSNA-fluorescent points (Figure 14, Figure 15 and Figure 16), while those of the intermediate neoplastic cell-type corresponded at both cytoplasmic SSNA-fluorescent points (Figure 17 and Figure 19), and nuclear SSNA-fluorescent points (Figure 19); their cytoplasmic SSNA-fluorescent points being quite close to the non-fluorescing cisternal structures. Those of the large neoplastic cell-type fairly corresponded at cytoplasmic SSNA-fluorescent points, close to the non-fluorescing cisternal structures (Figure 20, Figure 21 and Figure 22). Since cytoplasmic SSNAs are most likely to be RNA molecules in the form of ribonucleoproteins of ribosomes or m-RNAs and t-RNAs, their abundance within the three neoplastic cell-types compared to the normal lymphocytes with mostly nuclear SSNA lends supports to the concept of higher metabolic activity of the neoplastic cells vis-à-vis the normal cells. Based on the level of similarity of the positions of the highest pixel intensity points, as well as semblance in the outline of the curves of frequency distribution of pixel intensities, it may be possible to classify cells in general through such attributes. Classification of cells, based upon the uniqueness of their curves of frequency distribution of pixel intensities of nucleic acid fluorescent images, as well as concordance of points of highest pixel intensity values, could offer a cost-effective means for determination of changes between normal cells and their neoplastic counterparts. The rationale for this proposition stems from the fact that certain recognized biomarkers, such as DNA methylation reactions, simply reflect epigenetic changes on the genomic apparatus of the cell [19]. Since epigenetic hypermethylation and hypomethylation respectively results in a tighter (heterochromasia) and loosened (euchromasia) states of the chromatin, as reported in the literature [20-22], it seems likely that variation of the curves of pixel frequency distribution of the nucleic acid and concordance of highest points of pixel intensity values can be relied upon as an indicator or biomarker for the cellular changes. Such simple biomarker system can be applied towards cost-effective, routine diagnoses and prognostic monitoring of therapeutic interventions of neoplastic conditions in general. It is to be noted, however, that our findings at this stage are preliminary and still speculative; howbeit they could form reasonable basis for further work to be pursued for development of a reliable and cost-effective diagnostic and prognostic biomarker procedure for neoplastic cells based on their nucleic acid distribution pattern.

## Methods

Blood samples were collected from a total of 7 dogs, 3 of which were clinically normal with no history of any neoplastic condition, while 2 of the dogs were clinically confirmed for acute canine lymphocytic leukemia, and the remaining 2 for chronic lymphocytic leukemia. The leukemia cases were independently presented to our Veterinary Teaching Hospital at separate occasions. Diagnosis was based on observed clinical signs, history, and hematological features of the lymphocytes in the smears stained with Wright-Giemsa stain. Thin blood smears (6 slides per dog) were prepared; of the 6 slides smears per animal, half was processed for staining according to the standard Wright-Giemsa method (Benjamin, 1978), while the remaining half was stained with acridine orange (AO) stain for fluorescence microscopy viewing. Prior to staining, smears were air-dried, at room temperature, for at least 24 hrs in a dust-free incubator compartment, and subsequently fixed in 95% ethanol for 2 minutes.

### AO Staining Procedure

The AO staining procedure was performed according to a slightly modified Von-Bertallanfy *et al*. method (1958) for viewing with a Riechert epifluorescence microscope, interfaced with a high resolution megapixel Olympus DP12 digital image acquisition camera. The slightly modified Von-Bertallanfy *et al*. method involved hydration of the smears in distilled water for 5 seconds, brief rinsing in 1% acetic acid, brief rinsing in distilled water, and staining in 0.01% AO solution (pH 6.0, color index 788) for 3 mins. The stained smears were rinsed in 0.06 M phosphate buffer (pH 6.0) for 1 min, and air-dried in a dark dust-free compartment. Fluorescence microscopy viewing was accomplished with the aid of the epifluorescence microscope, with a mercury lamp source generating blue-spectrum excitation incidence beam.

### Image Digitalization and Analysis

All acquired 24 bits BMP fluorescent images were pre-processed by conversion into 8-bit grayscale images, with the aid of Corel Photo Paint^® ^standard graphic processing software. These pre-processed images were saved as uncompressed tagged image format (TIF). Further processing of the 8 bits grayscale TIF images was accomplished with the aid of a standard digital imaging software called TimWin^®^. The algorithms of the software collectively implement functions ranging from cellular logic bit plane pixel manipulations, to lookup table (LUT) color mapping operations of the pixels.

Digital imaging analytical procedure involved determination of spot pixel intensity values of the 8-bit gray tone images, through direct placement of the cursor over the image, and subsequent subtraction of background pixel intensity values (noise). An addition of a constant pixel intensity value equal to the difference between the maximum image pixel intensity value, following the background pixel value subtraction, and a set reference pixel intensity value of 100 was performed. Thus all processed images, free of background pixels or noise, had a maximum pixel intensity value of 100. The segmented images were captured by means of the Corel Capture utility^®^. Curves of frequency distribution of pixel intensity values of representative neoplastic and normal lymphocytes were generated with the aid of the TimWin^® ^software, and comparatively evaluated. Details on the logistics of the digital analytical method are available in our earlier reports [23,24].

Statistical analysis of the mean pixel intensity values of pixel population around the highest pixel reference value of 100, for the processed images, was performed by one-way analysis of variance (ANOVA) test, employing standard computer software statistical module. The mean pixel values of an equal count (n = 135) of pixel intensity values of 4 (normal lymphocytes) and 3 images for each neoplastic cell-type were used in the statistical analysis.

## Competing interests

The authors declare that they have no competing interests.

## Authors' contributions

GNI did the bulk of the study and write-up, including fluorescence microscopy and digital image analysis. MC was responsible for the clinical pathological evaluation and reports. SBN assisted with obtaining the references, specimen collection and proof reading. All authors read and approved the final manuscript.
